# Mapping the oxygen structure of γ-Al_2_O_3_ by high-field solid-state NMR spectroscopy

**DOI:** 10.1038/s41467-020-17470-4

**Published:** 2020-07-17

**Authors:** Qiang Wang, Wenzheng Li, Ivan Hung, Frederic Mentink-Vigier, Xiaoling Wang, Guodong Qi, Xiang Wang, Zhehong Gan, Jun Xu, Feng Deng

**Affiliations:** 1 National Centre for Magnetic Resonance in Wuhan, State Key Laboratory of Magnetic Resonance and Atomic and Molecular Physics, Key Laboratory of Magnetic Resonance in Biological Systems, Wuhan Institute of Physics and Mathematics, Innovation Academy for Precision Measurement Science and Technology, Chinese Academy of Sciences, Wuhan, 430071 China; 2 National High Magnetic Field Laboratory, 1800 East Paul Dirac Drive, Tallahassee, FL 32310–3706 USA; 3 University of Chinese Academy of Sciences, Beijing, 100049 China; 4 Wuhan National Laboratory for Optoelectronics, Huazhong University of Science and Technology, Wuhan, 430074 China

**Keywords:** Heterogeneous catalysis, Physical chemistry

## Abstract

γ-Al_2_O_3_ is one of the most widely used catalysts or catalyst supports in numerous industrial catalytic processes. Understanding the structure of γ-Al_2_O_3_ is essential to tuning its physicochemical property, which still remains a great challenge. We report a strategy for the observation and determination of oxygen structure of γ-Al_2_O_3_ by using two-dimensional (2D) solid-state NMR spectroscopy at high field. 2D ^17^O double-quantum single-quantum homonuclear correlation NMR experiment is conducted at an ultra-high magnetic field of 35.2 T to reveal the spatial proximities between different oxygen species from the bulk to surface. Furthermore, 2D proton-detected ^1^H-^17^O heteronuclear correlation NMR experiments allow for a rapid identification and differentiation of surface hydroxyl groups and (sub-)surface oxygen species. Our experimental results demonstrate a non-random distribution of oxygen species in γ-Al_2_O_3_.

## Abstract (web summary)

γ-Al_2_O_3_ is widely used in catalytic processes, but understanding its detailed structure remains a challenge. The authors, using two-dimensional solid-state NMR spectroscopy at a high magnetic field, characterize the spatial proximity and connectivity between oxygen species from the bulk to the surface.

## Introduction

Owing to their specific physical and chemical properties, metal oxides are of great importance in the field of materials and chemical science as diverse as electronics, energy storage and catalysis. In particular, the variability of their structure/phase and localized electronic structures has motivated widespread applications of metal oxides in heterogeneous catalysis^1–6^. γ-Al_2_O_3_ is widely used in a broad range of industrial catalytic reactions such as alcohol dehydration, propane dehydrogenation, isomerization, alkylation and catalytic cracking^7^. Optimization and rational design of related heterogeneous catalysts rely on detailed knowledge of the structure–property relationship. Over the past decades, considerable efforts have been devoted to experimental and/or theoretical explorations of the structure and nature of γ-Al_2_O_3_, which however remain poorly understood^8–17^. Unambiguous determination of the location of Al and O atoms is hampered by the large line-broadening of reflections/diffuse scattering pattern of small γ-Al_2_O_3_ particles in X-ray diffraction (XRD), neutron diffraction (ND) and electron diffraction characterizations. The surface species, particularly the coordinatively unsaturated atoms in γ-Al_2_O_3_ are usually correlated with particle size, morphology and pretreatment conditions^18–20^. The oxygen speciation (e.g., hydroxyl or defects) of γ-Al_2_O_3_ impacts its surface properties (i.e., acidity/basicity)_,_ and in many cases the proposed catalytic mechanisms are closely related to the local environment of oxygen atoms^21^. Thus, determination of oxygen species in γ-Al_2_O_3_ is prerequisite for understanding its structure and physicochemical property, which has remained a great challenge.

Solid-state NMR spectroscopy is a powerful and versatile tool for structural characterization of materials in either ordered or disordered states via the measurement of nuclear spin interactions^22–26^. ^17^O magic-angle spinning (MAS) NMR has been utilized in recent years to probe the local environments of oxygen atoms in various oxygen-containing materials^27–32^. However, a combination of quadrupolar nature for ^17^O nucleus (I = 5/2), relatively low gyromagnetic ratio (*γ* = −5.774 MHz T^−1^), and low ^17^O abundance (0.037%) leads to great difficulties using ^17^O NMR to investigate oxide-based materials especially at conventional magnetic fields (≤14.1 T). Therefore, the acquisition of ^17^O MAS NMR spectrum often entails ^17^O isotopic enrichment^33^. Additionally, advanced NMR methods and techniques are required to further enhance sensitivity and resolution. High-resolution two-dimensional (2D) experiments, such as ^29^Si-^17^O heteronuclear correlation (HETCOR) and ^17^O multiple-quantum (MQ) MAS experiments were reported in the structural characterization of inorganic materials (e.g., zeolite)^34^. Taking advantage of dynamic nuclear polarization (DNP) technique^35–38^, Pruski and coworkers showed the direct observation of Brønsted acid sites at natural abundance by ^17^O DNP surface-enhanced NMR spectroscopy (SENS) on silica-alumina samples. Since continuous distribution of ^17^O chemical shifts result in poor resolution of ^17^O MAS NMR spectrum, the broad ^17^O signals were analyzed by observing the change of their lineshapes associated with different hydroxyl environments (aluminols, silanols or Al(OH)Si)^39^. Our recent work showed that the surface oxygen sites with (e.g., aluminols and adsorbed water) or without (bare oxygen) bound protons on γ-Al_2_O_3_ can be detected by ^17^O DNP SENS through 2D ^17^O MQMAS and ^1^H-^17^O HETCOR experiments^40^. However, the determination of spatial proximity/connectivity between different oxygen sites has not been achieved yet, which is essential to better understand the local structure of γ-Al_2_O_3_. The conventional NMR approach is hampered by the small ^17^O-^17^O interactions due to the low γ of the ^17^O nucleus and the dilution from insufficient ^17^O isotope enrichment.

The oxygen speciation and its local structure in γ-Al_2_O_3_ remain largely unknown. The emerging high magnetic field up to 35.2 T (1.5 GHz) with dramatically improved sensitivity and resolution opens new stage for the application of ^17^O NMR spectrosopy^41–43^. In this contribution, we propose a strategy to unambiguously determine oxygen structure of γ-Al_2_O_3_ with the aid of ultrahigh magnetic field and state-of-the-art pulse sequences. To the best of our knowledge, the 2D ^17^O-^17^O double quantum (DQ)–single quantum (SQ) homonuclear correlation spectrum of γ-Al_2_O_3_ is for the first time acquired at magnetic field of 35.2 T. Even for the γ-Al_2_O_3_ sample with moderate ^17^O labeling (ca. 20%), the 2D correlation maps can be achieved in several hours. 4-coordinated and 3-coordinated oxygen sites are identified and their spatial proximities between different oxygen species from the bulk to surface are revealed. In combination with 2D proton-detected ^1^H-^17^O heteronuclear multiple quantum correlation (HMQC) experiments at 18.8 T, the discrimination of (sub-)surface oxygen species including bare oxygen, hydroxyl groups and adsorbed water is achieved. 3-coordinated oxygen sites are preferentially formed on the (sub-)surface of γ-Al_2_O_3_. Our ^17^O NMR experimental results demonstrate a non-random distribution of oxygen species and a non-spinel structure in γ-Al_2_O_3_. The detailed insights into the oxygen sites provide a basis for tuning the property of γ-Al_2_O_3_ and rational design of improved oxide-based catalysts.

## Results

### Identification of oxygen sites by 2D ^17^O 3QMAS NMR

Efficient enrichment of ^17^O is essential for ^17^O NMR spectroscopy especially the 2D correlation spectroscopy to study the structure of oxides. ^17^O-enriched γ-Al_2_O_3_ (BET surface area, 139.8 m^2^/g) was prepared with H_2_^17^O (40%, ^17^O) and boehmite. The labeled ^17^O atoms in γ-Al_2_O_3_ is less than 21% with respect to the total oxygen atoms in the sample (see Methods section for details). XRD and TEM image of the ^17^O-enriched γ-Al_2_O_3_ show the crystalline phase and morphology (see Supplementary Fig. 1 and Fig. 2). The IR data show the presence of different types of hydroxyl groups on γ-Al_2_O_3_ (Supplementary Fig. 3), which are not influenced by ^17^O isotope labeling treatment. The expected 4- and 6-coordinated Al (Al_IV_ and Al_VI_) sites along with a small amount of 5-coordinated Al (Al_V_) on the sample are revealed by ^27^Al MAS NMR (Supplementary Fig. 4a). Raising dehydrated temperature from 473 to 673 K leads to a slight decrease of the Al_VI_ accompanying with an increase of Al_V_ (Supplementary Fig. 4a). Therefore, the formation of Al_V_ can be related to the dehydroxylation of surface Al_VI_, and a relatively higher treatment temperature would result in more Al_V_ on the surface γ-Al_2_O_3_. It is noteworthy that there are many factors such as preparation method, morphology, particle size and post-treatment, could contribute to the variability of the surface property of γ-Al_2_O_3_^16,18,20,21,24^.

**Fig. 1:**
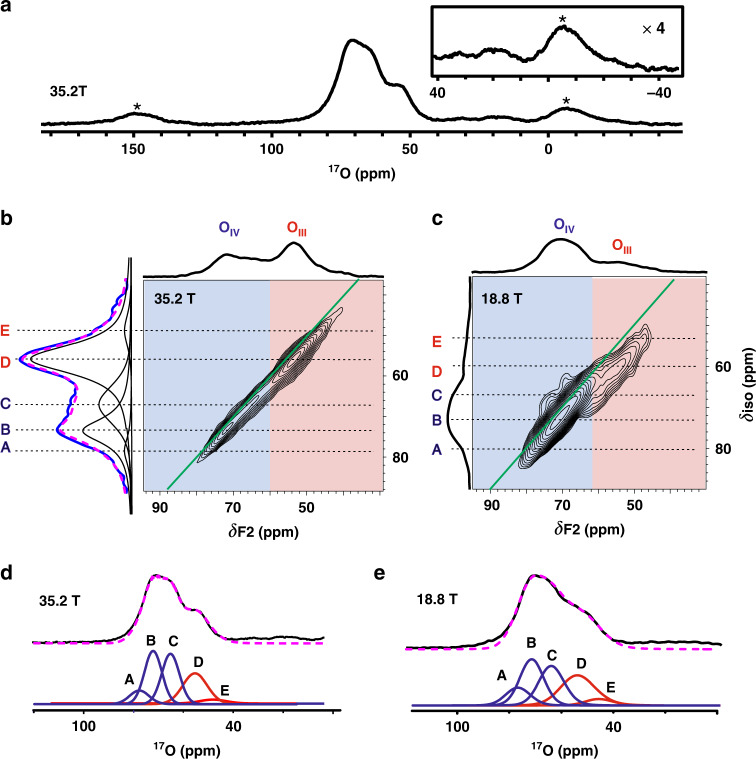
Identification of oxygen sites by 1D ^17^O MAS NMR and 2D ^17^O 3QMAS NMR. **a** 1D ^17^O MAS NMR spectrum of γ-Al_2_O_3_ acquired at 35.2 T and partially enlarged ^17^O spectrum, **b** sheared 2D ^17^O 3QMAS spectrum of γ-Al_2_O_3_ recorded on 35.2 T and projection (blue) in isotropic dimension with the simulated spectrum (purple) deconvoluted by five individual Gaussian lines (black, A-E denote as five individual oxygen sites), **c** sheared 2D ^17^O 3QMAS spectrum of γ-Al_2_O_3_ recorded at 18.8 T. Green lines in 2D spectra represent chemical shift/diagonal axis (*C*_Q_ = 0). O_IV_ and O_III_ denote 4-coordinated and 3-coordinated oxygen sites, respectively. **d**, **e** The best-fit simulation spectra (purple) of the 1D ^17^O MAS NMR spectra (black) of γ-Al_2_O_3_ obtained at 35.2 T and 18.8 T. The 1D spectra are deconvoluted with the DMFIT program^57^ according to the NMR parameters of five oxygen sites (A-E) in Table S1. The acquisition times of the 2D ^17^O 3QMAS spectra at 35.2 T and 18.8 T are ca. 0.53 h and ca. 40 h, respectively. Asterisks denote spinning sidebands.

The ^17^O MAS NMR spectrum of ^17^O labeled γ-Al_2_O_3_ dehydrated at 473 K was acquired at 35.2 T. As shown in Fig. 1a, a main signal centered at ca. 71 ppm and high-field shoulders (ca. 50~65 ppm) are observed, which can be assigned to 4-coordinated (OAl_4_, denoted as O_IV_) oxygen sites and 3-coordinated (OAl_3_, denoted as O_III_) oxygen sites^15,44^, respectively. The high-field signals at ca. −10~40 ppm are resolved as well, which are broadened and indistinguishable at lower magnetic field of 18.8 T due to the low concentration and quadrupolar broadening (Supplementary Fig. 4b). These weak signals can be assigned to the surface hydroxyl groups^40^. In order to make a clear determination of these oxygen species, 2D triple-quantum (3Q) MAS experiments were conducted at 35.2 T and 18.8 T, respectively (Fig. 1b, c). The two overlapped resonances from the O_IV_ (O(Al)_4_) and O_III_ (O(Al)_3_) sites in the 1D ^17^O MAS NMR spectrum become well resolved in the 2D 3QMAS spectra, which allows for assignment of different types of oxygen species by their chemical shifts. The distribution of chemical shift rather than the quadrupolar broadening dominates the lineshape of the ^17^O NMR spectrum at high field of 35.2 T, hence it enables to deconvolute the isotropic projection of the 2D ^17^O 3QMAS by using five individual Gaussian lines (sites A-E in Fig. 1b). Sites B, C and D can be easily identified, while A and E are not well discriminable owing to their low concentrations. To further probe these oxygen sites, 2D ^17^O 3QMAS spectrum was also recorded at lower field of 18.8 T, in which the quadrupolar interactions of different oxygen sites could lead to discontinuous evolution of the chemical shift distribution (Fig. 1c). Besides sites B, C and D, sites A and E become well resolved by the cross-sections in the 2D spectrum. The existence of oxygen A and E is further confirmed by the following 2D ^17^O DQ-SQ homonuclear correlation and ^1^H {^17^O} HMQC experiments (see the next sections). The 3QMAS experiment does not provide significant spectral resolution, which can be account for by the facts including relatively small quadrapolar coupling constant (*C*_Q_), very high magnetic field (*B*_0_) and broad chemical shift distribution for multiple sites in γ-Al_2_O_3_. Nevertheless, the deviation from the line with *C*_Q_ = 0 (the green line in Fig. 1b, c) reflect how large the quadrupole shift is and allow for more precise determination of the chemical shift by subtracting or taking the quadrupole shift into account. Supplementary Table 1 lists the NMR parameters of all the oxygen species extracted from the 2D ^17^O 3QMAS spectra at two fields. Accordingly, the 1D ^17^O MAS NMR spectra obtained at the two fields can be well fitted by the five oxygen sites (Fig. 1d, e).

Distortions in the geometry of O(Al)_n_ (*n* = 3–4) sites lead to the variations for *P*_Q_ (quadrupole interaction product) and *δ*_cs_ (isotropic chemical shift) values (see Supplementary Table 1), as observed in the case of γ-alumina^15^, glasses^45^ and zeolites^34^. In comparison, O_III_ sites D-E exhibit larger quadrupolar couplings (2.7–3.1 MHz) than that of O_IV_ sites A-C (1.8–2.4 MHz). This can be understood by the fact that O_III_ sites experience larger distortions of local environment induced by cation vacancies or surface defects in γ-Al_2_O_3._ Since the high-field signals (ca. −10–40 ppm) from hydroxyl groups are too weak to be observed in the 2D 3QMAS spectra, all these resolved signals come from the oxygen species without bound protons.

### Determination of oxygen-oxygen structures by 2D ^17^O DQ-SQ experiment

**Fig. 2:**
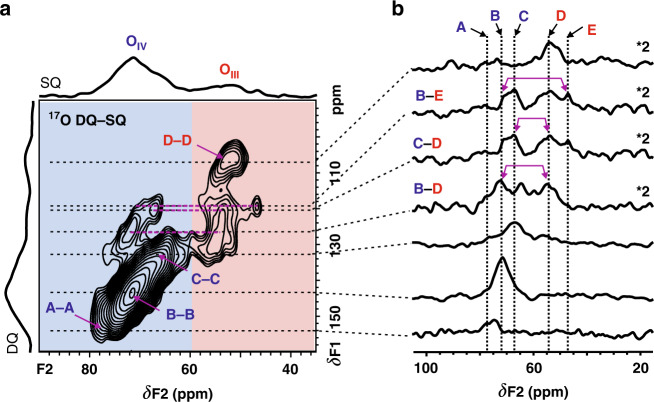
Probing oxygen-oxygen proximities via 2D ^17^O double quantum (DQ)- single quantum (SQ) experiments. **a** 2D ^17^O MAS DQ-SQ homonuclear correlation NMR spectrum of γ-Al_2_O_3_ recorded at 35.2 T and **b** extracted slices along F2 dimension. The acquisition time for the 2D spectrum is ca. 3.7 h. O_IV_ and O_III_ denote 4-coordinated and 3-coordinated oxygen sites, respectively. A-E denote five individual oxygen sites, A-A, B-B, C-C and D-D represent the auto-correlations from the same oxygen sites, B-E, C-D and B-D represent the cross-correlations from two distinct oxygen sites. The purple arrows in **b** indicate the position of two coupled oxygen sites in the extracted slices.

Up to now, there is no report yet on detecting the spatial proximity/connectivity of oxygen species in γ-Al_2_O_3_ largely due to insufficient ^17^O enrichment, detection sensitivity and spectral resolution required to correlate two low-γ S = 5/2 spins. For a specific nucleus, high magnetic fields lead to increase of its Larmor frequency and the net magnetization from the population difference between the neighboring energy levels (e.g., 1/2 ↔ −1/2), thus enhancing NMR signal through both resonance frequency and the polarization. More importantly for quadrupole nuclei, high fields reduce the broadening from the second-order quadrupolar effect, therefore provide a gain of spectral resolution proportional to the square of B_0_ in units of ppm^46^. With the aid of our developed BR2^1^_2_ pulse sequence^47^ and the dramatic sensitivity and resolution enhancement achieved by the Series-Connected Hybrid magnet capable of generating magnetic field of up to 35.2 T^42^, 2D ^17^O DQ-SQ homonuclear correlation spectrum was obtained on γ-Al_2_O_3_ sample in less than 4 h (Fig. 2a). Despite a low radio-frequency recoupling field (ca. 2.7 kHz) was used in the 2D ^17^O DQ-SQ NMR experiment, our previous work^47^ has demonstrated that the BR2^1^_2_ recoupling employed here was sufficient to cover a bandwidth of ca. 8 kHz (i.e., 45 ppm–80 ppm at 35.2 T). To the best of our knowledge, this is the first report of the 2D ^17^O DQ-SQ homonuclear correlation spectrum, which provides a direct view of the dipolar interactions between different types of oxygen species and thus of their spatial proximity.

Since the dipolar interaction is inversely proportional to the cube of the distance between two ^17^O atoms, the detected correlations in Fig. 2a should mainly originate from 2-bond ^17^O-Al-^17^O correlation rather than 4-bond ^17^O-Al-O-Al-^17^O correlation by considering the transfer efficiency as well as the dipolar truncation effect of the homonuclear recoupling^48^. Moreover, simulations were performed to compare DQ-SQ experimental efficiencies between isolated 2-bond and 4-bond ^17^O pair (Supplementary Fig. 5) based on the non-spinel γ-Al_2_O_3_ model^11^. The simulated double-quantum filtering (DQF) efficiency of the former remains over ten times higher than that of the latter at the point of experimental DQ recoupling time (*τ*_*re*_ = 3 ms) although the dipolar truncation effect is not taken into account. This suggests the detected correlations in Fig. 2a should mainly originate from 2-bond ^17^O-Al-^17^O correlation.

Four self-correlation peaks (A-A, B-B, C-C, and D-D) are observed along the diagonal line with a slope of 2, indicating the same types of the oxygen sites are in close proximity one another. While the cross peaks B-E, C-D and B-D reflect the spatial proximities between different oxygen species, which could be unambiguously identified in the 1D slices extracted from the 2D spectrum (Fig. 2b). Although the correlation peaks mainly depend on the strength of dipolar coupling (O-Al-O distance), the concentration of the proximate oxygen pairs contribute to the relative intensities as well. As shown in the 1D ^17^O MAS spectrum (Fig. 1) and 2D ^17^O DQ-SQ NMR spectrum (Fig. 2a), the signal B is more intense than the others. Thus, site B is most likely present as bulk species in γ-Al_2_O_3_. This is further supported by the fact that site B correlates with the other high-field O_III_ sites (D and E) (Fig. 2b). For the O_III_ site D, there is a downfield shift (1~2 ppm) of the center of gravity in its cross peaks (e.g., B-D) compared with its self-correlation peak (D-D). This implies that the slight difference of the oxygen-oxygen local structure in γ-Al_2_O_3_ might be an important factor influencing the distribution of electric field gradients (EFG) of oxygen sites, which yields a distribution of apparent chemical shifts of ^17^O NMR involving the isotropic quadrupolar shift. Note that an off-diagonal signal (ca. 78 ppm in F2 dimension) at the lowest field seriously overlaps with self-correlation peaks A-A and B-B. This off-diagonal correlation could be either the correlation between two A sites with continuous chemical shift distributions caused by slightly different local environments or the cross correlation between A and B. No additional cross-peak is observable with respect to site A, indicating that either this type of oxygen is not located in close proximity to other oxygen sites or there are less proximate oxygen pairs between site A and other oxygen sites. In addition, there is no apparent cross correlation between two distinct O_IV_ species (e.g., B-C, A-C) in the 2D ^17^O DQ-SQ spectrum (Fig. 2a), which suggests that different magnetically inequivalent O_IV_ sites are separated. Similarly, no cross correlation between site D and E observed in Fig. 2a reveals that these two O_III_ species are not in close proximity. These experimental results suggest that different oxygen species (magnetically inequivalent) discriminated here are closely related to their special O-Al-O local structures. Taking the information obtained from the 2D DQ-SQ NMR experiment together, it provides the first direct evidence that these oxygen sites in γ-Al_2_O_3_ exist in a non-random manner.

### Discrimination of surface oxygen species by 2D ^1^H{^17^O} HMQC experiment

Note that it’s not straightforward to correlate the surface oxygen species with that in the bulk by above ^17^O 3QMAS and DQ-SQ experiments since the surface oxygen species that are in form of hydroxyl groups are not observed in the 2D NMR spectra (Figs. 1 and 2). By taking advantage of localized interactions between protons and oxygen, ^1^H-^17^O HETCOR experiment could provide selective information on the surface hydroxyl groups and even the surface bare oxygen species provided the proton source such as hydroxyl group or adsorbed water are in close proximity to the target oxygen sites. However, the use of conventional ^1^H-^17^O cross polarization (CP) or ^17^O{^1^H} D-HMQC NMR experiment is time-consuming for quadrupolar nucleus with low γ and at low natural abundance^49,50^. Owing to the high sensitivity of ^1^H nuclei, proton-detected HETCOR experiments such as HMQC^51^, R-INEPT^32^ and TEDOR^43^ have proved to be robust methods to indirectly detect the insensitive nuclear spin as well as to explore its connectivity/proximity with proton under fast MAS. Since a single-resonance 3.2 mm probe was equipped on the 35.2 T NMR system which could not render fast MAS and double-resonance experiment, Therefore, we conducted the proton-detected 2D ^1^H {^17^O} *J*/D-HMQC experiments using a 1.9 mm double-resonance probe on a commercial at 18.8 T NMR spectrometer with a MAS speed of 40 kHz. The ^1^H-^17^O *J*-HMQC through-bond experiment allows for identification of hydroxyl groups (-OH) exposed on γ-Al_2_O_3_, while the 2D ^1^H-^17^O D-HMQC through-space experiment was used to probe bare oxygen (without H bonded) in close proximity with hydroxyl groups. The through-space method enables to detect bare ^17^O not only on the surface but also below the surface (denoted as sub-surface) provided there are similar internuclear distances (or dipolar interactions) between these bare oxygens and protons of surface hydroxyls.

**Fig. 3:**
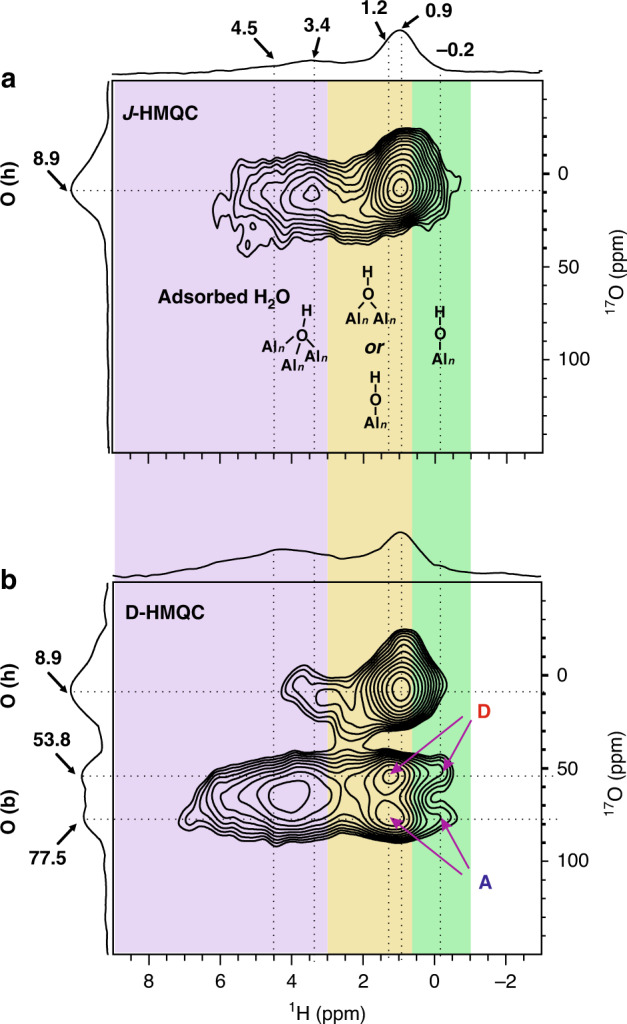
Discrimination of surface oxygen species by 2D ^1^H{^17^O} HMQC experiments. **a** 2D ^1^H{^17^O} *J*-HMQC spectrum and **b** 2D ^1^H{^17^O} D-HMQC spectrum of γ-Al_2_O_3_ with *τ*_re_ = 1.05 ms recorded at 18.8 T. The background colors differentiate the hydroxyl groups based on their isotopic chemical shifts in the ^1^H dimension. The oxygen species associated with hydroxyls and the bare oxygen species are denoted as O(h) and O(b), respectively. The structure of triply bridging, doubly bridging and terminal hydroxyls are displayed in Fig. 3a based on the distribution of respective ^1^H chemical shifts. A and D indicate the specific 4-coordinated and 3-coordinated oxygen sites, respectively, which are correlated with doubly bridging and terminal hydroxyls in Fig. 3b. The acquisition time for the *J*- and D-HMQC spectra is ca. 4.6 and 9.1 h, respectively.

According to previous experimental and theoretical reports^16,39^, the O-H bond length on γ-Al_2_O_3_ is about 1 Å with the corresponding *J* coupling of ca. 80 Hz. The 2D proton-detected ^1^H {^17^O} *J-*HMQC spectrum in Fig. 3a shows the chemical bond correlations through scalar-coupling transfer in which only the ^1^H-^17^O correlations from hydroxyl groups are observable. Although the ^17^O signals from different hydroxyl groups are severely overlapped in the ^17^O dimension, the distinguishable ^1^H signals help assign the hydroxyl groups on the surface of γ-Al_2_O_3_. The surface hydroxyl groups have been extensively examined by ^1^H solid-state NMR spectroscopy^18,20,24,52^. Three main spectral regions were identified at around 0 ppm, 1-3 ppm and 3-5 ppm, which fall into the chemical shift range of terminal (μ_1_) hydroxyl, doubly bridging (μ_2_) hydroxyl and triply bridging (μ_3_) hydroxyl groups, respectively. While the very recent work by Raybaud^20^ indicated that besides the exposed main surface of γ-Al_2_O_3_, the stepped surfaces and edges architectures should be taken into account to interpret the ^1^H NMR as well. Specifically, the −0.2 ppm ^1^H signal comes from the μ_1_-OH; the proton 0.9 and 1.2 ppm signals can be assigned to the μ_2_-OH or μ_1_-OH, while the signal at 3.4 ppm is ascribed to the μ_3_-OH. Besides, chemically adsorbed H_2_^17^O is observed on the sample surface reflected by the broad correlations at ^1^H chemical shift larger than 4.0 ppm, which is confirmed by the dramatic decrease of the corresponding ^1^H signal on the sample when dehydration temperature is raised from 473 K to 673 K (Supplementary Fig. 4c). The 2D ^1^H{^17^O} *J*-HMQC experiment directly reveals different types of hydroxyl groups through directly connecting oxygen with proton atoms. These surface hydroxyl groups confirm the observation of the weak high-field ^17^O signals in the ^17^O MAS NMR spectrum (Fig. 1a). Compared with the 1D ^1^H MAS NMR spectrum, a significant decline in the ^1^H signals (ca. 4.5 ppm) from adsorbed H_2_O is observed in the 2D ^1^H{^17^O} *J*-HMQC, implying much faster transverse relaxation of protons in the residual water molecules than that in hydroxyl groups on the surface of γ-Al_2_O_3_. Meanwhile the stronger intensity of the correlation peak in the 2D spectrum suggests that the μ_2_-OH dominates the surface hydroxyl groups on our γ-Al_2_O_3_ samples.

**Fig. 4:**
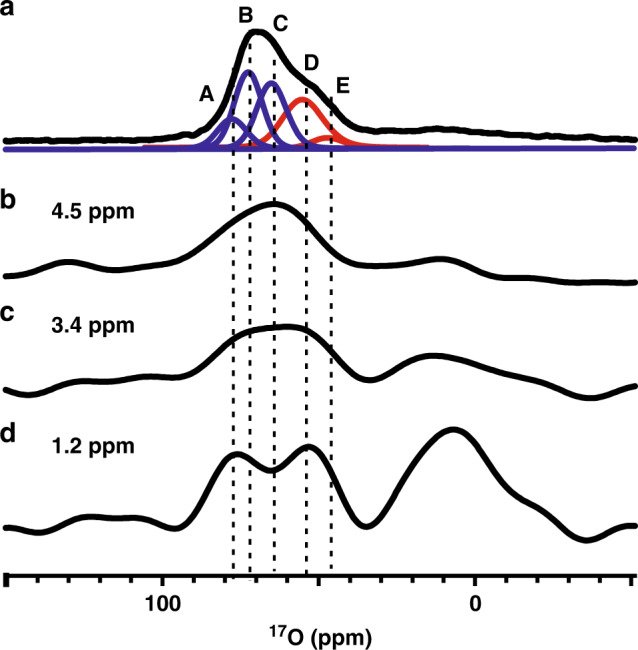
Comparison of specific ^17^O NMR slices extracted from Fig. 3b. Deconvolution lines of 1D ^17^O MAS NMR spectrum at 18.8 T (**a**), and extracted ^17^O NMR slices from 4.5 ppm (**b**), 3.4 ppm (**c**) and 1.2 ppm (**d**) in the ^1^H dimension of 2D ^1^H{^17^O} D-HMQC spectrum (Fig. 3b), respectively. Letters A-C denote three fitted 4-coordinated oxygen sites (blue lines), D-E denote two fitted 3-coordinated oxygen sites (red lines).

The ^1^H{^17^O} *J*-HMQC spectrum shows detailed information about chemical connectivity between surface oxygen and protons. In order to get extended picture of the surface oxygen structure, 2D ^1^H{^17^O} D-HMQC experiments were also conducted on γ-Al_2_O_3_. The ^1^H{^17^O} D-HMQC experiment uses the dipolar interaction to create correlation signal, which is more robust than the commonly used CP/MAS sequence for the investigation of spatial proximity between quadrupolar and spin-1/2 nuclei^53^. The variation of the recoupling time allows to probe local environment of the surface oxygen at various distances to hydroxyl protons. The ^1^H{^17^O} D-HMQC spectrum of γ-Al_2_O_3_ with a short recoupling time of 0.30 ms shows correlations to the neighboring species at short distance (Supplementary Fig. 6), similar to those achieved by the *J*-based experiment. Increasing the recoupling time to 1.05 ms gives rise to additional signals in Fig. 3b. Besides the ^1^H-^17^O correlations from different bridging hydroxyl groups (^17^O chemical shift at ca. −10–40 ppm), strong correlations between the protons of hydroxyl groups and the ^17^O signals at ca. 50–75 ppm are observed. The well-resolved low-field ^17^O signals can be assigned to oxygen site A and D, respectively, by considering their apparent ^17^O chemical shifts (*δ*_F2_). Figure 4 displays the selective 1D slice of the ^17^O NMR spectra along different ^1^H chemical shifts (1.2 ppm, 3.4 ppm and 4.5 ppm) extracted from the 2D ^1^H-^17^O D-HMQC spectrum. The correlation peaks indicate that the μ_1_-OH and μ_2_-OH groups (−0.2–2.0 ppm) are mainly located in close proximity to O_IV_ site A and O_III_ site D (also see Fig. 4d). Although it is difficult to differentiate ^17^O sites from the 1D ^17^O slices along the ^1^H signals at 3.4 ppm (Fig. 4c) and 4.5 ppm (Fig. 4b), the lineshape of the ^17^O slice for the latter implies that water molecule (4.5 ppm) is preferentially adsorbed on O_IV_ site C, showing its strong hydrophilicity on the surface of γ-Al_2_O_3_. While the intramolecular ^1^H-^17^O correlations for chemically adsorbed H_2_^17^O are hard to be observed, which can be accounted for by the relatively faster relaxation of the nuclear spins in water molecule during the period of dipolar recoupling. Note that different ^1^H species have distinct influences on the intensities of ^1^H-^17^O correlations in 2D HMQC experiment due to their different relaxations. We analyzed the ^1^H-^17^O correlation slices extracted from specific ^1^H resonances (i.e., 4.5 ppm, 3.4 ppm and 1.2 ppm in ^1^H dimension, see Fig. 4). For a specific ^1^H resonance, its transverse relaxation would attenuate its ^1^H-^17^O correlations on similar level in 2D HMQC spectra, which would thus does not significantly influence the relative intensities of distinct ^17^O species in its ^1^H-^17^O correlation slice in the ^17^O dimension.

No apparent correlations are observed between protons and oxygen site B in the 2D D-HMQC spectrum. This is a clear indication that this oxygen species should be not in close proximity to the surface protons. The longer internuclear distance makes the dipolar interaction too weak to be detected by the D-HMQC experiment. Taking the results together, it strongly suggests that site B exist in the bulk while sites A, C and D are most likely present on the (sub-)surface of γ-Al_2_O_3_.

The influence of the heating treatment on the distribution of the surface oxygen species on γ-Al_2_O_3_ was analyzed by ^1^H{^17^O} D-HMQC. ^17^O MAS NMR (Supplementary Fig. 4b) shows that there is an apparent decrease of the ^17^O signals from the hydroxyl groups (ca. −10–45 ppm) along with the increase of the ^17^O signals from bare oxygen species (ca. 45–80 ppm) on γ-Al_2_O_3_ when the dehydration temperature is raised from 473 to 673 K. Interestingly, the ^1^H-^17^O correlations revealed by the 2D ^1^H{^17^O} D-HMQC spectrum of γ-Al_2_O_3_ dehydrated at 673 K (Supplementary Fig. 7) are quite similar to those observed on the sample dehydrated at lower temperature (Fig. 3b). In particular, the residual μ_2_-OH and μ_1_-OH (−0.2–2.0 ppm) on γ-Al_2_O_3_ keep close proximity to the specific O_IV_ (e.g., site A) and O_III_ (e.g., site D) species. This indicates that heating treatment at mild condition does not have significant impact on the structure of the surface oxygen species on γ-Al_2_O_3_.

**Fig. 5:**
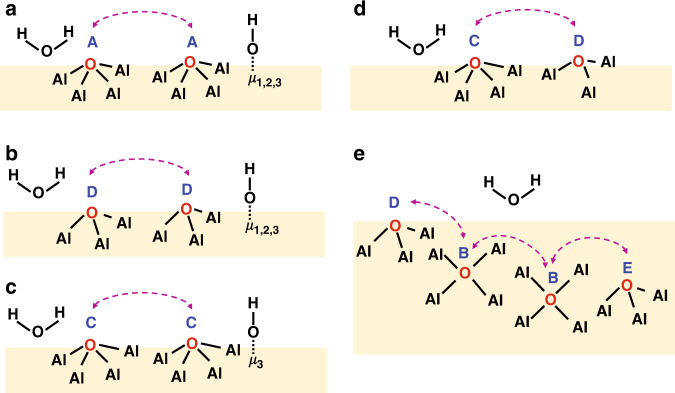
Proposed local structure models of oxygen species in γ-Al_2_O_3_. **a**–**e** Letters A-E represent five distinct bare oxygen sites. The purple dotted arrows indicate the spatial proximities between two specific bare oxygen sites. Surface terminal (μ_1_), doubly (μ_2_) and triply (μ_3_) bridging hydroxyl groups are indicated.

The detailed information about the oxygen speciation and spatial proximities and connectivities of different oxygen sites allows us to propose local structure models of γ-Al_2_O_3_ (Fig. 5). The (sub-)surface of γ-Al_2_O_3_ is occupied by different types of oxygen sites (A, C and D) which are in close proximity to surface hydroxyl group or water molecule (Fig. 5a–c). The O_IV_ (site C) and O_III_ (site D) species are found to be located in close proximity as well (Fig. 5d). In spite of the low concentration, oxygen site E that is mostly likely in the bulk can still be identified based on its spatial proximity with oxygen site B (Fig. 5e). While the bulk oxygen site B is probably located near to the surface of γ-Al_2_O_3_ as evidenced by its proximity to site D.

## Discussion

The complex oxygen species in γ-Al_2_O_3_ in terms of speciation and distribution often bring about difficulty in the characterization of their structures and properties. This is demonstrated by the overlapping ^17^O signals and insufficient spectral resolution even in the 2D ^17^O 3QMAS spectrum recorded at high field (Fig. 1b, c). The high-resolution cross peaks provided by the 2D ^17^O DQ-SQ and ^1^H {^17^O} HMQC spectra enables to resolve and determine these overlapped components. This makes the quantitative measurements of distinct oxygen species feasible on the 1D ^17^O NMR spectra (Fig. 1d, e).

The ^17^O NMR spectra allow to not only obtain unprecedented information on the local environment of oxygen species but also shed insight into the structure of γ-Al_2_O_3_ by analyzing the location of Al^3+^ vacancies^17^. In the spinel-like model, the vacancy at an octahedral position results in the formation of six O_III_ anions. While the vacancy at a tetrahedral position produces four O_III_ anions, leading to a non-spinel structure^11^. Therefore, the γ-Al_2_O_3_ bulk structure could be determined via a quantitative analysis of O_IV_ and O_III_ sites in 1D ^17^O MAS NMR spectrum. For example, the O_III_/O_IV_ ratio in a unit cell of Al_8_O_12_ is 1.0 or 0.5 for an Al^3+^ vacancy at octahedral or tetrahedral position, respectively. The deconvolutions of 1D ^17^O MAS NMR spectra of γ-Al_2_O_3_ (Fig. 1d, e) assisted by the improved resolution of the 2D ^17^O DQ-SQ and ^1^H {^17^O} D-HMQC spectra clearly show an O_III_/O_IV_ ratio of 0.51 ± 0.03 at 18. 8 T or 0.45 ± 0.04 at 35.2 T (Supplementary Table 1), respectively, suggesting that Al vacancies should be located at tetrahedral position. In addition, ^27^Al MAS NMR spectroscopy allows for the determination of the location of the Al vacancies in γ-Al_2_O_3_ by measuring the proportion of octahedral Al_VI_ or tetrahedral Al_IV_ sites. If the vacancies (V) are at octahedral positions, γ-Al_2_O_3_ is expressed as Al_IV_[Al_5/3_V_1/3_]_VI_O_4_ and 62.5% Al^3+^ cations are located at octahedral positions^17^. For the vacancies at tetrahedral positions, γ-Al_2_O_3_ gives a the formula of [Al_2/3_V_1/3_] _IV_[Al_2_]_VI_O_4_ and there are 75% Al^3+^ cations at octahedral positions. The 1D ^27^Al MAS spectrum of γ-Al_2_O_3_ dehydrated at 473 K (Supplementary Fig. 4a) shows that 73% of Al^3+^ cations is in octahedral coordination, confirming that the vacancies are mainly at tetrahedral positions. This is also in agreement with previous ^27^Al DQ-SQ experimental result on γ-Al_2_O_3_^54^, where there is no correlation from two coupled tetrahedral ^27^Al observed in the 2D ^27^Al homonuclear correlation spectrum. It should be noted that the thermal treatment impacts to some extent the coordination of Al atom in γ-Al_2_O_3_. As shown in Supplementary Fig. 4a, the 6-coordinated Al decreases from 73% to 70% upon increasing the dehydration temperature from 473 to 673 K. Note that there is a small variation (from 67.5% to 75%) of Al_VI_ upon varying vacancies from octahedral to tetrahedral Al. This would lead to a larger measurement error on the amount of vacancies at tetrahedral or octahedral positions in ^27^Al MAS NMR compared with that obtained in 1D quantitative ^17^O MAS NMR. Nevertheless, the Al vacancies are predominately located at tetrahedral positions, which is a clear indication of the non-spinel structure for γ-Al_2_O_3_.

In our previous DNP study of γ-Al_2_O_3_^40^, surface-selectively labeling was performed by surface exchange of commercial γ-Al_2_O_3_ with ^17^O_2_ gas or H_2_^17^O. In addition to the surface hydroxyl groups, O_III_ and O_IV_ species were observed. Aiming at a detailed understanding of oxygen species in γ-Al_2_O_3_, herein we investigated the labeled samples both on the surface and in the bulk which were prepared by dehydration of ^17^O-enriched boehmite. The thermal treatment would influence more or less the oxygen species particularly on the surface of γ-Al_2_O_3_. For example, the surface hydroxyl groups were obviously decreased with increasing of dehydration temperature, which was confirmed by our 1D ^17^O and ^1^H MAS NMR spectra (Supplementary Fig. 4b and 4c). In spite of the variation of surface species, the 2D ^1^H-^17^O D-HMQC spectrum revealed the preferential formation of O_III_ species on the (sub-)surface of γ-Al_2_O_3_ (Fig. 3b), in agreement with the result obtained by the direct ^17^O DNP spectroscopy^40^.

The basic property of γ-Al_2_O_3_ is related to surface oxygen sites. For example, the terminal surface hydroxyl group was found to have selective reactivity toward CO_2_^24^. Some active sites (so called defect sites) are supposed to be formed by the Lewis acid (tri-coordinated Al)–base (O) pairs on the metastable surface of γ-Al_2_O_3_^21^. Furthermore, the surface oxygen on γ-Al_2_O_3_ could serve as the anchoring site for metal supported catalysts. The distribution of the (sub-)surface oxygen especially the coordinately unsaturated oxygen such as site D led us to believe that the anchoring of the supported metal species on γ-Al_2_O_3_ would be non-random. The isolated or vicinal oxygen sites would influence the formation of metal ions or clusters, which have been demonstrated to exhibit distinct activity^55^.

In summary, we provide a robust and reliable ^17^O NMR strategy to map the oxygen structure in γ-Al_2_O_3_. The combined high magnetic fields and fast MAS demonstrate the ability to increase the sensitivity and resolution of 2D ^17^O MAS NMR spectra. Different oxygen species and their spatial proximities in γ-Al_2_O are identified by ^17^O 3QMAS in combination of 2D ^17^O DQ-SQ homonuclear correlation NMR experiments conducted at 35.2 T. The proton-detected ^1^H-^17^O *J*/D-HMQC experiments allow a rapid detection of the (sub-)surface oxygen species through chemical bond or spatial/dipolar interaction with surface protons including water and hydroxyl groups. The results presented herein not only provide unique insights into the structure of oxygen sites of γ-Al_2_O_3_, which has important implications for tuning the property of γ-Al_2_O_3_, but also demonstrate the ability to utilize the increased resolution/sensitivity of 2D ^17^O NMR experiments at high or ultra-high magnetic fields to investigate various complex metal oxide-based catalysts. The detailed information on oxygen speciation and its local structure would enable to better understand the anchoring site for supporting metals as well as the specific interaction between oxides surface and reaction molecules, which is helpful for the rational design of improved catalysts.

## Methods

### Preparation of ^17^O enriched γ-Al_2_O_3_

^17^O-enriched boehmite was first prepared by exchanging boehmite with H_2_^17^O. 380 μl (0.38 g) H_2_^17^O (^17^O, 40%) was introduced into a glass tube containing 500 mg pretreated AlOOH•H_2_O. The glass tube was sealed with a flame after degassing on a vacuum line at 423 K for 48 h. Then, ^17^O-enriched γ-Al_2_O_3_ was prepared by dehydration of the ^17^O-enriched boehmite at 773 K for 4 h. Consequently, the enriched γ-Al_2_O_3_ samples were dehydrated at 473 K or 673 K for 2 h and were stored in sealed glass tube to minimize adsorption of atmospheric moisture. The amount of ^17^O labeling can be roughly estimated. A total of~ 20 mmol of H_2_^17^O (^17^O, 40%) was used for 500 mg of AlOOH•H_2_O. The maximum labeled ^17^O atoms should be less than 21% ([20 mmol*40%(^17^O)]/[20 mmol(${\rm{O}}_{{\rm{H}}_{2}{\rm{O}}}$) + 19.5 mmol(${\rm{O}}_{{\rm{AlOOH•}} {{\rm{H}}_{2}{\rm{O}}}}$)]) with respect to the total oxygen atoms of the sample.

### Solid-state NMR measurements

1D ^17^O MAS NMR, 2D ^17^O 3QMAS and DQ-SQ MAS NMR spectra were recorded on 35.2 T series-connected hybrid magnet at the National High Magnetic Field Laboratory (NHMFL) using a Bruker Avance NEO console and a single-resonance 3.2 mm MAS probe designed and constructed at the NHMFL. A pulse width of 3 µs corresponding to a π/6 flip-angle and a recycle delay of 1 s were used to collect the single-pulse ^17^O MAS NMR spectrum of γ-Al_2_O_3_ at a spinning speed of 15.6 kHz. The number of transients collected was 128. The 2D ^17^O 3QMAS spectrum of γ-Al_2_O_3_ was collected using a shifted-echo sequence, and all pulse widths were optimized on the sample at a spinning speed of 15 kHz. A total of 48 scans were collected for each of the 40 rotor-synchronized *t*_*1*_ increments with a recycle delay of 1 s. 2D ^17^O DQ-SQ MAS NMR experiment was conducted using BR2^1^_2_ recouplings (*τ*_*re*_ = 3 ms), and all pulse widths were optimized at a spinning speed of 16 kHz. Central transition (CT) enhancement of the initial magnetizations (^17^O) was obtained by using a SS-WURST-80 irradiation with a length of 1 ms. A total of 832 scans were collected for each of the 16 rotor-synchronized *t*_*1*_ increments with a recycle delay of 1 s.

1D ^17^O MAS NMR and 2D ^17^O 3QMAS spectra were also recorded on 18.8 T with a MAS speed of 15 kHz by using a Bruker Avance III 800 spectrometer and a 3.2 mm double resonance probe. A pulse width of 1.3 µs corresponding to a π/6 flip-angle and a recycle delay of 0.5 s were used to collect the single-pulse ^17^O MAS NMR spectrum with 1600 scans. The 2D ^17^O 3QMAS spectrum of γ-Al_2_O_3_ was collected using a z-filtering sequence, and all pulse widths were optimized on the sample at a spinning speed of 15 kHz. A total of 2400 scans were collected for each of the 40 *t*_*1*_ increments (Δ*t*_*1*_ = 50 μs) with a recycle delay of 1.5 s.

1D single-pulse ^27^Al, ^1^H and ^17^O MAS NMR spectra were collected at 18.8 T with a MAS speed of 40 kHz by using a 1.9 m double-resonance probe. A pulse width of 1.85 µs corresponding to a π/2 flip-angle and a recycle delay of 10 s were used to collect the single-pulse ^1^H MAS NMR spectrum with 8 scans. A pulse width of 0.7 µs corresponding to a π/6 flip-angle and a recycle delay of 1 s were used to collect the single-pulse ^27^Al MAS NMR spectrum with 240 scans. A pulse width of 0.83 µs corresponding to a π/6 flip-angle and a recycle delay of 2 s were used to collect the single-pulse ^17^O MAS NMR spectrum with 1500 scans as well. Note that the small flip-angle single-pulse experiment was used for acquiring 1D ^17^O MAS NMR spectra, which is less susceptible to the relatively short recovery delays. In principle, any non-negligible chemical shift anisotropy (CSA) should be considered for quantitative analysis of the 1D ^17^O MAS NMR spectrum at high field. To this end, the ^17^O NMR spectra (Supplementary Fig. 8) were acquired and compared at two different MAS speeds (15 and 40 kHz) with two different relaxation delays (0.5 and 2 s respectively), which reveals that the relaxation and CSA do not have significant influence on our analysis.

2D ^1^H {^17^O} *J*-HMQC spectra of γ-Al_2_O_3_ were collected at 18.8 T with a MAS speed of 40 kHz by using a 1.9 m double-resonance probe. A total of 256 scans were collected for each of the 32 rotor-synchronized *t*_*1*_ increments with a recycle delay of 2 s. 2D ^1^H {^17^O} *D*-HMQC spectra of γ-Al_2_O_3_ dehydrated at 473 K were collected using SR4 recouplings with *τ*_*re*_ = 1.05 ms and 0.30 ms at a spinning speed of 40 kHz, respectively. A total of 512 scans (*τ*_*re*_ = 1.05 ms) and 128 scans (*τ*_re_ = 0.30 ms) were collected for each of the 32 rotor-synchronized *t*_*1*_ increments with recycle delays of 2 s. 2D ^1^H {^17^O} *D*-HMQC spectrum of γ-Al_2_O_3_ dehydrated at 673 K were collected using SR4 recouplings with *τ*_*re*_ = 1.05 ms at a spinning speed of 40 kHz. A total of 256 scans were collected for each of the 32 rotor-synchronized *t*_*1*_ increments with recycle delays of 4 s. Shorter relaxation delays were used in acquiring the 2D experiments for saving whole acquisition time due to the limit magnet time^42^. Pre-saturation pulses and/or satellite saturation pulses were employed to spoil residual longitude magnetizations at the beginning of each scan. For above 2D experiments, presaturation has been applied with a train of 10 π/2 pulses 20 ms apart.

The ^1^H transverse relaxation (T_2_) is measured by using a pseudo 2D experiment (chemical shift (CS)-echo) at 18.8 T with a MAS speed of 40 kHz. The T_2_ of the ^1^H signal from adsorbed water at 4.5 ppm is ca. 0.23 ms, almost half of that (ca. 0.41 ms) of Al-OH species at 1.2 ppm.

The chemical shifts were referenced to liquid H_2_O, which is 0 ppm for ^17^O and 4.8 ppm for ^1^H.

### Simulations

Simulations were performed with the SIMPSON software^56^, and the powder averaging was performed using 320 crystallites following the REPULSION algorithm. *C*_Q_ values of two coupled oxygen atoms are 2 MHz and 3 MHz, respectively.

### X-ray powder diffraction (XRD) experiments

XRD patterns were recorded on a Panalytical X’ Pert PRO X-ray diffractometer (40 kV, 40 mA) using CuKα (*λ* = 1.5406 Å) radiation. The scan rate is 0.05 degrees per second for XRD measurements.

### TEM measurement

TEM image was obtained on a HITACHI HT7700 instrument at an accelerating voltage of 100 kV.

### IR measurement

Diffuse reflectance Fourier transform infrared (DRIFT) spectra were acquired on a Bruker Tensor 27 spectrometer which equipped with CaF2 windows. The samples were packed into the DRIFT cell in a glovebox under dry nitrogen atmosphere. 128 scans were accumulated by a mercury-cadmium telluride (MCT) detector for each spectrum (resolution, 2 cm^−1^), and the background spectrum was collected by KBr.

## Supplementary information

Supplementary Information

## Data Availability

All relevant data are available from the authors.
