# Supplementary material for: Mapping the oxygen structure of γ-Al2O3 by high-field solid-state NMR spectroscopy
Source: Nat Commun. 2020 Jul 17;11:3620. doi: 10.1038/s41467-020-17470-4 (PMC7367832; doi:10.1038/s41467-020-17470-4)
Supplement: Supplementary file 1 — Supplementary Information [file 41467_2020_17470_MOESM1_ESM.pdf]

Supporting Information

Mapping the Oxygen Structure of γ-Al_2O_3 by High-Field Solid-state NMR Spectroscopy

Wang et al.

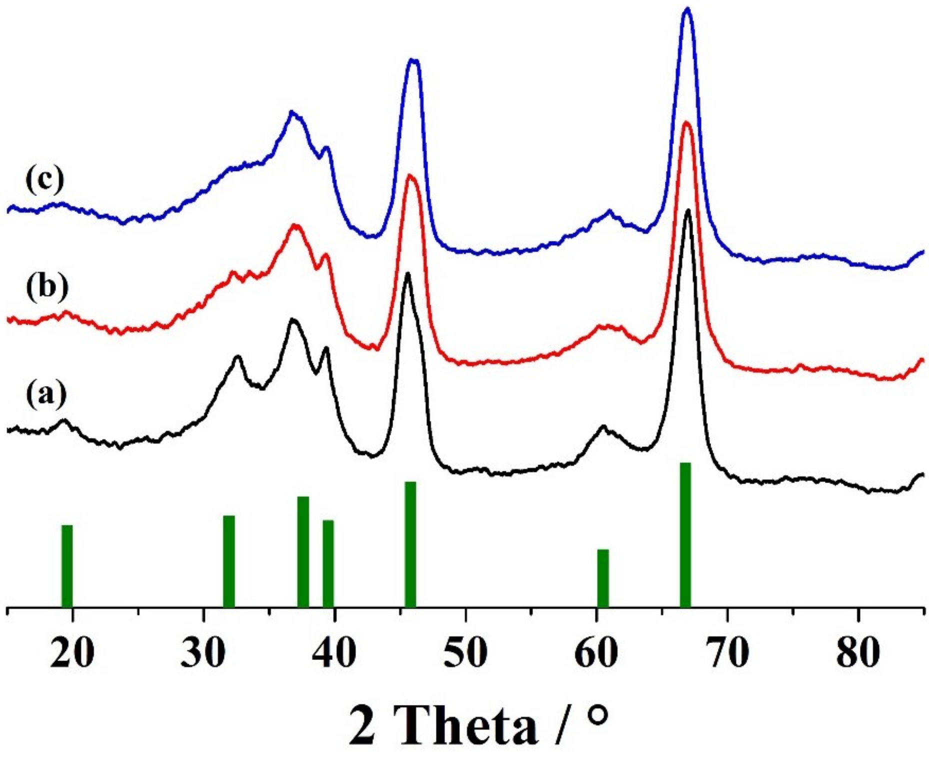

Supplementary Figure 1. XRD patterns of commercial (a), natural abundance (b), and ^{17}O-enriched (c) γ-Al_2O_3. The latter two samples were prepared with boehmite and ^{17}O-enriched boehmite (exchanged with $H_2{}^{17}O$) respectively.

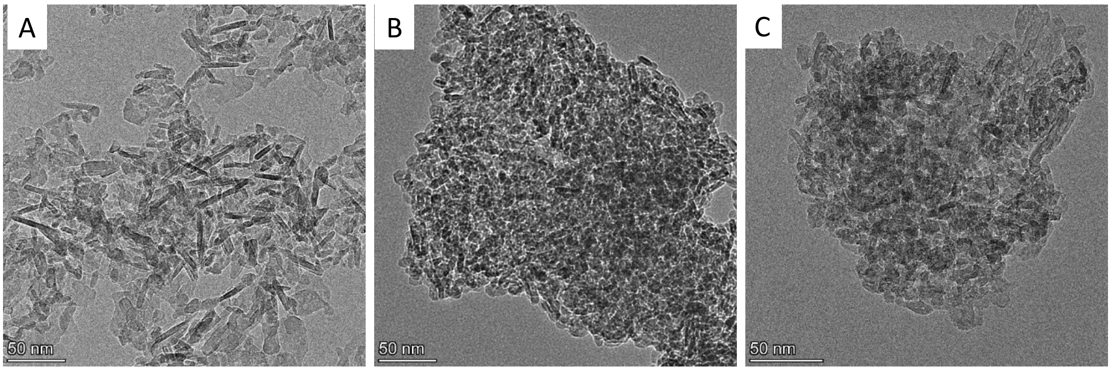

Supplementary Figure 2. TEM images of commercial (a), natural abundance (b) and ^{17}O-enriched (c) γ-Al_2O_3. The latter two samples were prepared by boehmite and ^{17}O-enriched boehmite (exchanged with $H_2{}^{17}O$) respectively.

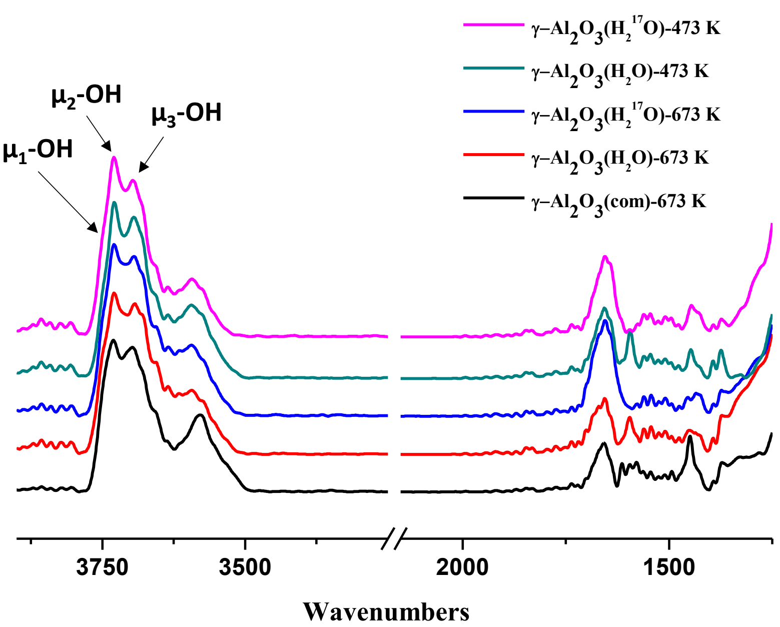

Supplementary Figure 3. DRIFT spectra of ^{17}O-enriched γ-Al_2O_3 dehydrated at 473 K (purple) and 673 K (green), natural abundance γ-Al_2O_3 dehydrated at 473 K (blue) and 673 K (red) and commercial γ-Al_2O_3 (black). The assignments are according to Knözinger et al.[1]

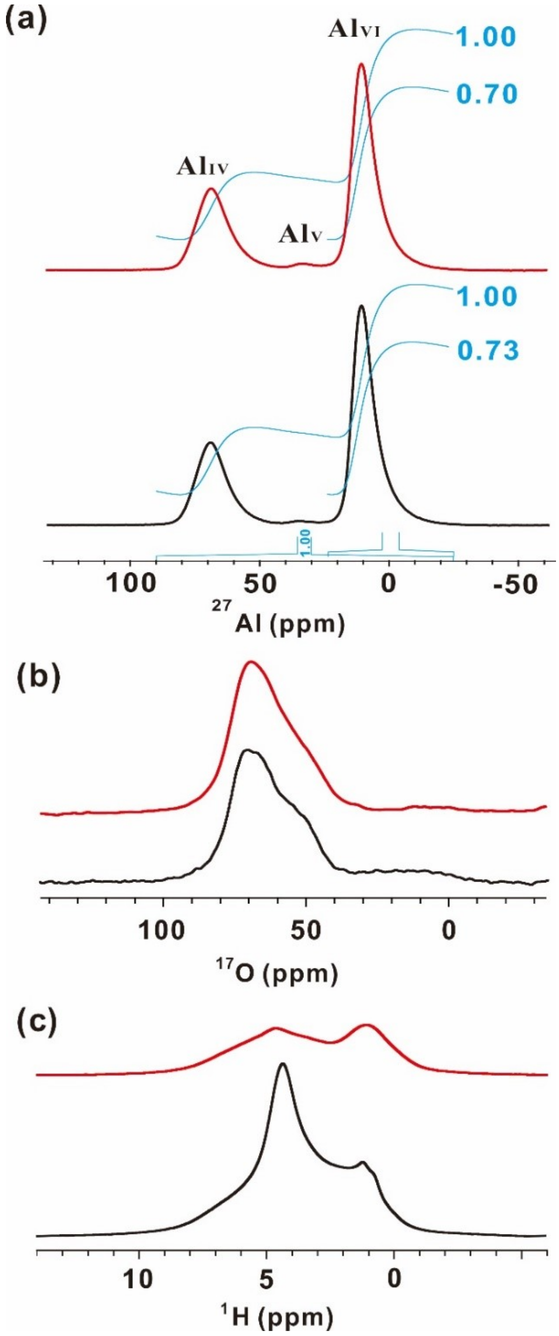

Supplementary Figure 4. 1D ^{27}Al (a), ^{17}O (b) and ^{1}H (c) MAS NMR spectra of ^{17}O-enriched γ-Al_2O_3 dehydrated at 473 K (black) and 673 K (red) acquired at 18.8 T. The blue curves in Fig. S4a represent different integral regions normalized to the region from 90 ppm to -25 ppm.

Supplementary Table 1. NMR parameters extracted from the ^{17}O 3QMAS spectra recorded at 35.2 T and 18.8 T, respectively. δ_{F2} represents the centre of gravity of each species measured along direct dimension (F_2). Isotropic chemical shift δ_{CS} and quadrupole interaction product $P_Q = C_Q\sqrt{1+\eta^2/3}$ of each oxygen species were derived from Fig. 1b and 1c, where C_Q is the quadrupolar coupling constant and η is the asymmetric parameter of the electric field gradient (EFG) tensor.

**^{17}O sites**		**36.0 T**				**18.8 T**			
		**δ_{F2}[a] (ppm)**	**δ_{CS}[b] (ppm)**	**P_Q[b] (MHz)**	**Percent[c,d] (%)**	**δ_{F2}[a] (ppm)**	**δ_{CS}[b] (ppm)**	**P_Q[b] (MHz)**	**Percent[c,d] (%)**
O_{IV}	**A**	77.5 ± 0.8	78.1 ± 0.6	2.1 ± 0.6	8	77.5 ± 1.0	79.2 ± 0.7	1.8 ± 0.3	11
	**B**	71.9 ± 0.5	72.5 ± 0.4	2.1 ± 0.4	32	70.3 ± 1.0	71.9 ± 0.7	1.8 ± 0.3	29
	**C**	65.7 ± 1.0	66.5 ± 0.7	2.4 ± 0.7	29	63.0 ± 2.0	65.6 ± 1.5	2.2 ± 0.4	26
O_{III}	**D**	54.1 ± 0.8	55.4 ± 0.6	3.0 ± 0.4	27	53.8 ± 2.0	57.4 ± 1.5	2.7 ± 0.4	28
	**E**	46.8 ± 1.0	48.3 ± 0.7	3.2 ± 0.5	4	45.0 ± 1.5	49.6 ± 1.1	3.0 ± 0.2	6

a. A small measurement error of δ_{F2} could cause a large deviation of the calculated P_Q at such high field, thus we calculated the uncertainty (error bar) of δ_{F2} from multiple measurements.

b. Error bars of P_Q and δ_{CS} (uncertainty in indirect measurements) were derived according to the transfer law of uncertainty

c. Relative intensity of each fitted oxygen species. For simulations, the initial C_Q and δ_{F2} values were set based on the NMR parameters. According to the formula $P_Q = C_Q\sqrt{1+\eta^2/3}$, C_Q parameters were obtained with the asymmetric parameter $\eta = 0.7$ by taking intermediate value of C_Q when varying η between 0 and 1. Since the dominated chemical shift distribution results in the absence of typical quadrupolar pattern in the ^{17}O MAS NMR spectrum, it is impossible to extract the η of individual resonance. A line broadening of 1.10 ppm was used to smooth the discontinuities stemming from the sampling of C_Q when simulations were performed using the Czjzek model implemented in the DMFit program, which should be at the same level of precision compared with uncertainties of NMR parameters.

d. The error analysis was conducted for the calculation of the O_{III}/O_{IV} ratio based on the signal-to-noise ratio of the 1D ^{17}O MAS NMR spectra

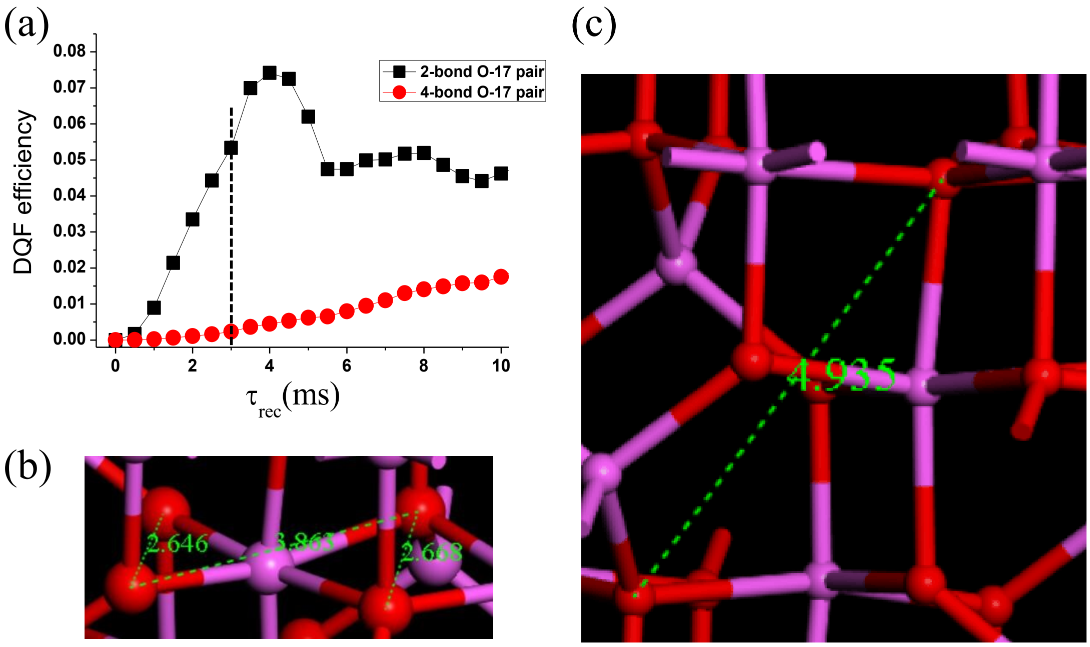

Supplementary Figure 5. (a) Simulated double-quantum filtering (DQF) efficiencies of isolated 2-bond (black square) and 4-bond (red circle) ^{17}O spin pairs, with ^{17}O-^{17}O distances being 2.65 Å and 4.94 Å, respectively. Local structure of γ-Al_2O_3 with (b) 2-bond and (c) 4-bond O-O distances being indicated.[2]

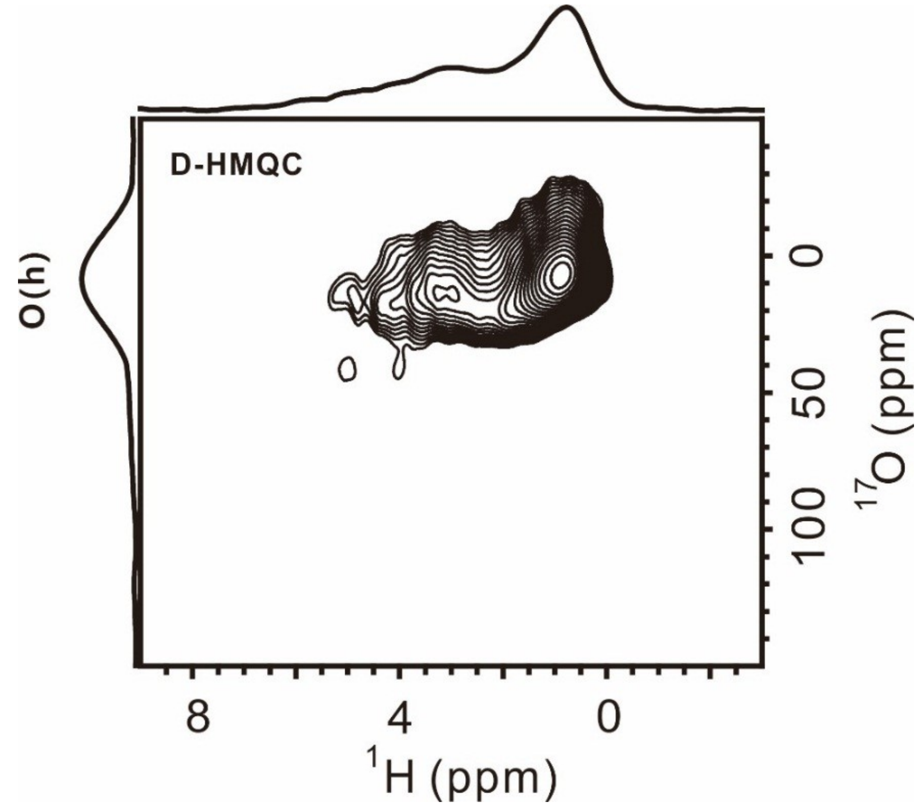

Supplementary Figure 6. ^{1}H {^{17}O} D-HMQC spectrum of ^{17}O-enriched γ-Al_2O_3 acquired at 18.8 T with τ_{re} = 0.30 ms and a spinning speed of 40 kHz. The acquisition time is ca. 2.3 h. O(h) denotes the oxygen species associated with hydroxyls.

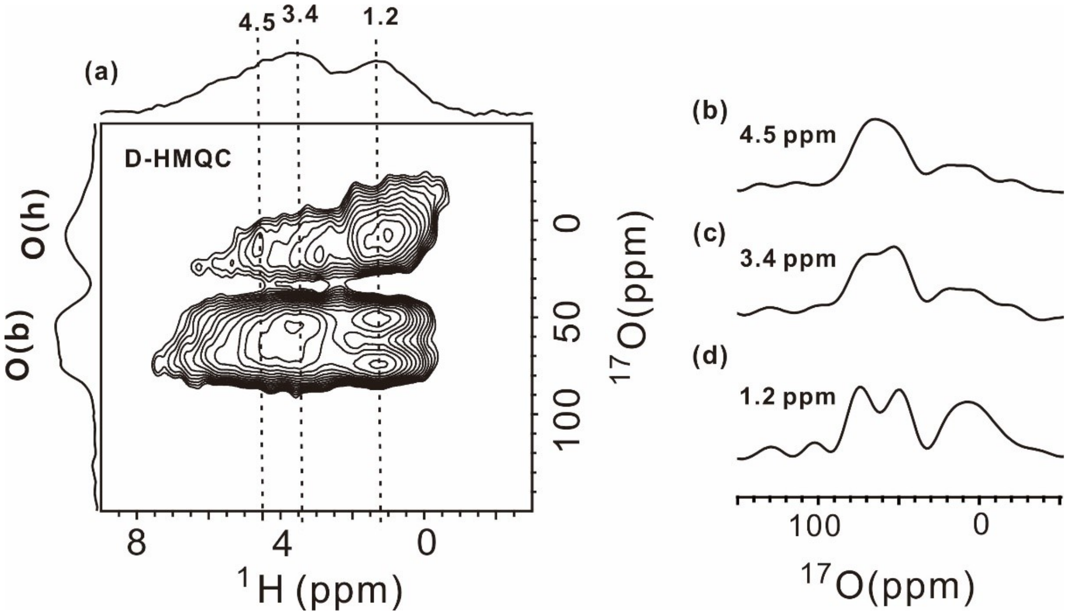

Supplementary Figure 7. 2D $^1H\{^{17}O\}$ D-HMQC spectrum of ^{17}O-enriched γ-Al_2O_3 (dehydrated at 673 K) acquired at 18.8 T with τ_{re} = 1.05 ms (a) and extracted ^{17}O NMR slices from 4.5 ppm (b), 3.4 ppm (c) and 1.2 ppm (d) in the ^1H dimension of the 2D spectrum. The oxygen species associated with hydroxyls and the bare oxygen species are denoted as O(h) and O(b), respectively.

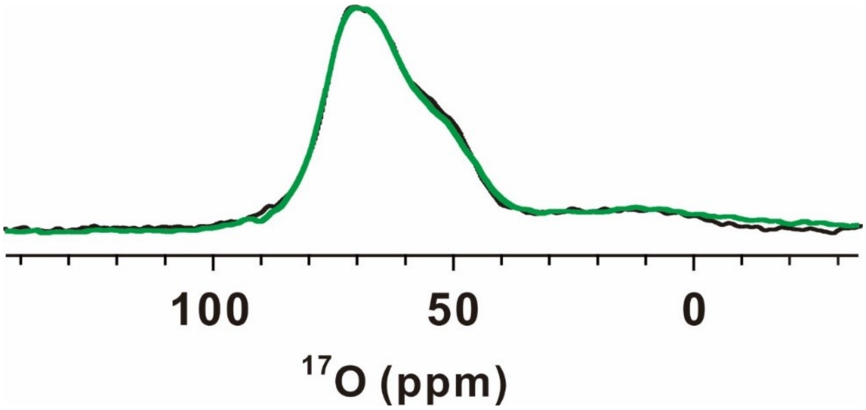

Supplementary Figure 8. 1D single-pulse ^{17}O MAS NMR spectra of ^{17}O-enriched γ-Al_2O_3 dehydrated at 473 K acquired at 18.8 T with a MAS speed of 15 kHz and a relaxation delay of 0.5 s (black) and with a MAS speed of 40 kHz and a relaxation delay of 2.0 s (green).

Supplementary References

[1] Knözinger, H.; Ratnasamy, P., Catalytic Aluminas: Surface Models and Characterization of Surface Sites. *Catal. Rev. Sci. Eng.* 17, 31-70 (1978).

[2] Digne, M.; Sautet, P.; Raybaud, P.; Euzen, P.; Toulhoat, H., Use of DFT to achieve a rational understanding of acid–basic properties of γ-alumina surfaces. *J. Catal.* 226, 54-68 (2004).
